# Strong coupling of metamaterials with cavity photons: toward non-Hermitian optics

**DOI:** 10.1515/nanoph-2023-0899

**Published:** 2024-02-05

**Authors:** Fanqi Meng, Lei Cao, Juliette Mangeney, Hartmut G. Roskos

**Affiliations:** Physikalisches Institut, Johann Wolfgang Goethe-Universität, Frankfurt am Main, Germany; State Key Laboratory of Advanced Electromagnetic Technology, Huazhong University of Science and Technology, Wuhan 430074, China; École normale supérieure (Paris), Paris, France

**Keywords:** metamaterials, strong coupling, non-Hermitian

## Abstract

The investigation of strong coupling between light and matter is an important field of research. Its significance arises not only from the emergence of a plethora of intriguing chemical and physical phenomena, often novel and unexpected, but also from its provision of important tool sets for the design of core components for novel chemical, electronic, and photonic devices such as quantum computers, lasers, amplifiers, modulators, sensors and more. Strong coupling has been demonstrated for various material systems and spectral regimes, each exhibiting unique features and applications. In this perspective, we will focus on a sub-field of this domain of research and discuss the strong coupling between *metamaterials* and *photonic cavities at THz frequencies*. The metamaterials, themselves electromagnetic resonators, serve as “artificial atoms”. We provide a concise overview of recent advances and outline possible research directions in this vital and impactful field of interdisciplinary science.

## 1 Introduction

In free space, when photons interact with a material and excite electrons from the ground state to a higher energy state, the electrons – if non-radiative relaxation channels are inaccessible – naturally return to the ground state through spontaneous emission. This process is irreversible, with the emission rate dependent on vacuum electromagnetic wave fluctuations represented as continuous optical modes in free space. Modification of the surrounding environment can significantly alter the vacuum field and, consequently, the spontaneous emission rate. Under specific conditions, when the material is positioned within a photonic cavity, spontaneous emission can be reversed, leading to the re-absorption and re-emission of the emitted photon. This process induces Rabi oscillations, where the electron oscillates between ground and excited states at the angular Rabi frequency 2*g*, where *g* is the strength of coupling between the dipole moment of the material excitation and the respective cavity mode [1]. The system enters the strong-coupling regime when the coupling strength exceeds the decay rate (*κ* + *γ*)/2 of the excitation determined by the decay rate *κ* of the excited state in the material and that of the photons in the cavity *γ*. Investigating strong coupling lies at the core of cavity quantum electrodynamics (cavity QED) [2].

The exploration of cavity QED began with Rydberg atoms [2] and was later extended to various (quasi-)particles, including interband (excitonic) or intersubband transitions in 2D materials and quantum well/dot structures [3], [4], [5], [6], cyclotron resonances in 2D electron gases [7], [8], magnon resonances [9], [10], and superconducting two-level systems [11], [12]. Over the last decade, there has been a renewed and even growing interest in investigating the strong coupling of molecules via their vibrational modes with cavity photons [13], [14].

While conventional investigations of cavity QED focus on extracting new photonic features from strong coupling [2], recent research has shifted towards understanding how strong coupling modifies material characteristics, such as enhancing conductivity/superconductivity and controlling chemical reactions involving excited and ground states [15], [16], [17], [18].

This paper considers metamaterials (MMs) as matter being placed into cavities. MMs are periodic structures with sub-wavelength features [19]. Their response to electromagnetic radiation can be manipulated by their structural design and material composition, making them interesting for numerous applications, namely in photonics and sensing. Research on MMs has flourished in its own right in the last two decades [20], [21], [22], [23]. Here, we mainly focus on planar metal-based MMs. Illumination with electromagnetic radiation leads to plasmonic resonances arising from the collective motion of the electrons in the micro- and nano-patterned metallic structures.

This perspective aims to summarize the state-of-the-art of strong coupling between plasmons, especially those in MMs, and photons in cavities. In addition, the emphasis is on an attempt to identify future developments in this emerging research direction.

## 2 Strong coupling of plasmons with cavity photons

**Figure 1:**
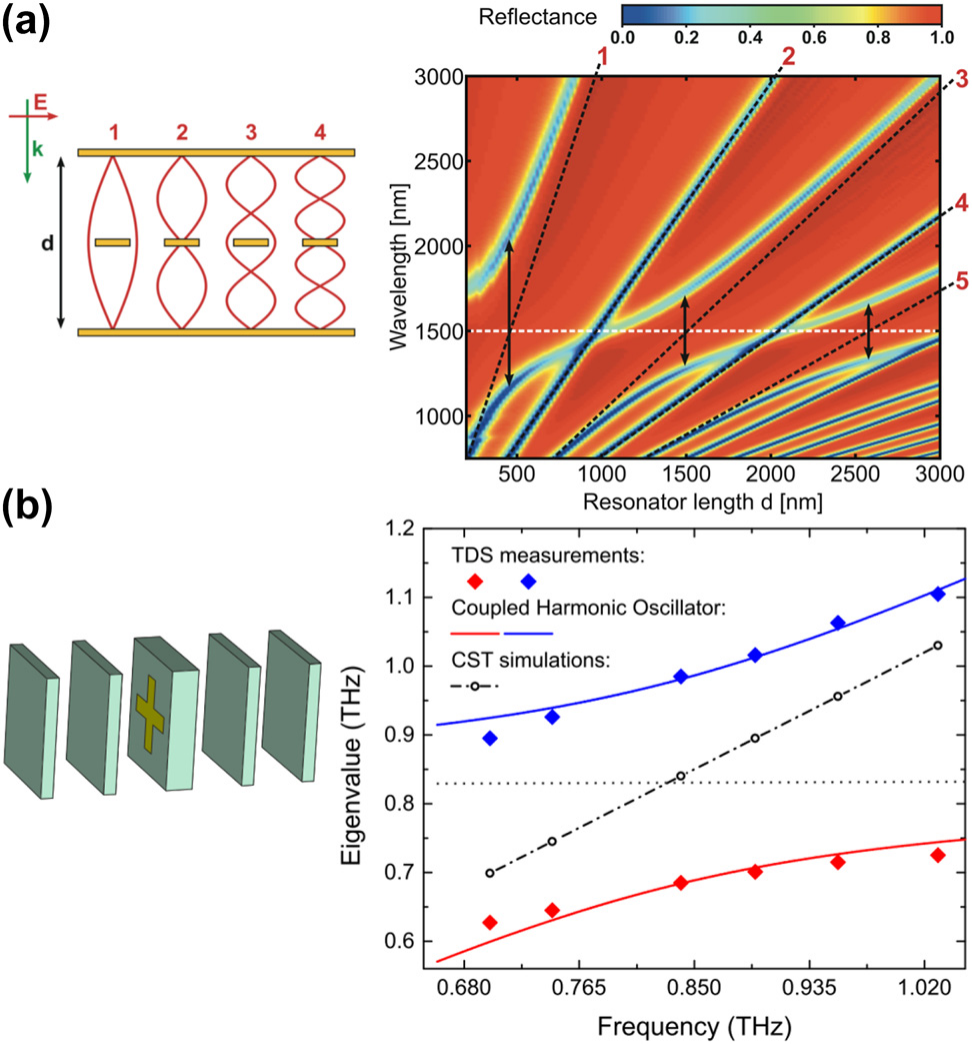
Two examples of plasmonic structures in a 1D photonic cavity. (a) Nanowire array in a Fabry–Perot cavity [29] (figures reproduced with permission from John Wiley and Sons). Left: Schematic view of the cavity with the nanowire sample in its center. Right: Infrared reflectance spectrum as a function of the length of the cavity. The dashed white and black lines correspond to the non-interacting plasmon and cavity modes. Around the intersection of the odd cavity modes with the local plasmon mode, an anticrossing is observable. (b) Swiss-cross MM in a 1D photonic-crystal cavity [30] (figures reproduced with permission from Optica). Left: Schematic view of a Swiss-cross unit cell of the two-dimensional MM placed on the surface of the cavity’s defect layer. Right: Calculated and measured dispersion of the upper/lower polariton modes. Diamonds represent the measured peak frequencies of various MM structures with different geometrical sizes in the cavity. The peak frequencies are plotted versus the theoretical absorption resonance frequency of each bare MM. Red and blue solid curves are calculated with a coupled-harmonic-oscillator model.

The early exploration of strong coupling involving plasmons concentrated on the visible and infrared spectral range. The small wavelength of the radiation brought about challenges with regard to the fabrication of the required structures. Advances in nanotechnology enabled the observation and manipulation of local and surface plasmons. The breakthrough came with the integration of a grating structure with an underlying waveguide [24]. The grating’s surface plasmon strongly coupled with the waveguide mode, featuring a Rabi splitting of 250 meV, approximately 12 % of the resonance frequency of the waveguide mode (with photon energy of 1.9 eV). Coupled systems of local or surface plasmons with waveguide modes then became platforms for the investigation of nanophotonic phenomena including the study of the temporal dynamics of the coupled modes [25], [26], [27]. Advancements in 3D nanofabrication enabled the integration of metallic nanowires and nanowire pairs into Fabry–Perot cavities [28], [29]. The left side of Figure 1(a) schematically shows a sample consisting of a planar array of nanowires in the center of a Fabry–Perot cavity. The right part of Figure 1(a) shows the measured reflectance spectrum as a function of the length *d* of the cavity. The spectrum exhibits anti-crossing features of the odd cavity modes (mode numbers 1, 3, 5) at such cavity lengths where the anti-node positions of the standing waves superimpose with the nanowire, thereby maximizing the coupling strength of the electric field of the mode with the electric dipole moment of the nanowire. Moreover, by integrating nanowire pairs into the cavity, the symmetric and antisymmetric localized plasmon modes of the pairs were shown to strongly couple with the electric field and magnetic field of the cavity, respectively [29].

Fabricating nanowires and Fabry–Perot cavities for the visible frequency range is technologically demanding. Not surprisingly, the less challenging terahertz frequency regime has recently seen more attention. Strong coupling was reported between localized surface plasmons of Swiss-cross and split-ring-resonator MMs and photons of a one-dimensional photonic crystal (1D PC) cavity at sub-1-THz frequencies [30]. The left side of Figure 1(b) shows the schematic of a unit cell of the two-dimensional Swiss-cross MM in the 1D PC cavity. The 1D PC was constructed from five air-gap-separated silicon slabs, a thicker one serving as the central defect layer and two outer pairs with identical thicknesses forming Bragg mirrors on both sides. All layers were made from slabs of electrically highly resistive silicon. A Rabi splitting of 2*g* = 300 GHz was reported, which accounts for 34 % of the cavity resonance frequency *f*_*c*_ of 860 GHz, thus representing a case of ultrastrong coupling (*g*/*f*_*c*_ > 0.1). The coupling strength exhibits a square-root dependence on the density of MM unit cells, indicating that all the unit cells of the MMs interact collectively with photons. In particular, this collective interaction can also occur in a nonlocal way between spatially separated MM layers in the cavity.

The availability of a wide family of MMs with different properties brings in rich proven functionalities to the coupling with cavities. An example is MMs optimized as sensors (using mode frequency shifts as the measured quantity) [31], [32], [33], [34]. The potential arising here for the MMs upon use in systems with ultrastrong coupling has not yet been explored much. One of the few examples in the literature is a theoretical study of the use of a split-ring-based MM in a 1D PC cavity for sensing purposes [35]. One finds that the shift of the resonance frequency of the polariton modes, induced by an analyte applied to the MM, is smaller in the case of the hybrid mode than the shift obtained with the MM alone. This perhaps counter-intuitive result finds its explanation in the increase of the mode volume by the cavity which is detrimental to the sensitivity [36]. The study considers, however, also the use of phase changes as measured quantity instead of frequency shifts. The sensitivity of the hybrid modes can then be larger than that of the MM’s plasmon resonance alone. This finding suggests a novel way to high-performance sensors.

Conventional planar MMs have their electric dipole moments oriented parallel to the substrate surface. They can be coupled readily with the electric field of the cavity. In contrast, the magnetic dipole moments of the MMs are perpendicular to the substrate and do not commonly interact with the magnetic field of waves impinging perpendicular to the MM. However, exploiting Babinet’s principle, it was shown that complementary MMs (CMMs) possess magnetic dipole moments that are parallel to the surface of the substrate [37]. Thus CMMs provide an avenue to couple with the magnetic field of the cavity [38]. Strong coupling between CMMs and cavity photons was reported using *half* cavities or Tamm cavities [39], [40], [41], [42], [43]. In such coupled systems, the polariton modes can exhibit very small mode volumes $\left(<10^{-4}\cdot \lambda^{3}\right)$ and relatively high values of the Q-factor (>35) [43].

Until now, we have only considered the simultaneous coupling of a single cavity mode with a single MM plasmonic mode. It is, however, possible to expand the number of involved modes. This was demonstrated by strong coupling between two plasmon modes and one cavity mode [43], but also by strong coupling between one plasmon mode and two cavity modes [39]. In the former case, the MM had a unit cell with two split-ring resonators of slightly different dimensions and resonance frequencies; in the latter case, a CMM coupled to two 1D PC cavities with different eigenmodes. Both scenarios led to the emergence of three polariton modes. It should be mentioned that these schemes can readily be extended to couple even larger numbers of modes. The emerging systems can be considered in some ways as classical equivalents of quantum-mechanical systems exhibiting many-particle interactions [43].

Another route to coupling of four modes was followed in an experiment, which involved dark modes of a MM. Such dark modes do not interact with incoming radiation. This can be the case because the field polarization and the orientation of the dipole moments of the MM do not match, or because the interaction of constituents of the MM’s unit cell leads to destructive interference and a vanishing net dipole moment. Dark modes of MMs have been extensively investigated to suppress radiative loss and realize different functionalities [8], [44], [45], [46]. For instance, by coupling dark plasmon modes with bright plasmon modes, plasmonic electromagnetically induced transparency (EIT), the occurrence of Fano resonances, and bound states in the continuum (BIC) were demonstrated [45], [47], [48]. The resonances in these cases normally possess very high Q-factors and can be employed for sensing and lasing applications [33]. In the four-mode-coupling experiment mentioned above, an EIT-like MM was integrated into a 1D PC cavity. The MM had a unit cell consisting of two split-ring resonators rotated by 90° relative to each other. This led to the existence of a bright and a dark plasmonic mode (’dark’ with respect to the incoming linearly polarized radiation). Because of their proximity, the modes were coupled to each other. The cavity exhibited a rotational symmetry with respect to the optical axis, which allowed for the existence of two equivalent degenerate cavity modes, one bright (being excited by the incoming radiation), and the other dark. In the integrated system, the coupling of the two plasmonic modes led to an interaction of all four modes, which revealed itself in the appearance of four polariton modes [43].

## 3 Perspective

The static strong interaction between plasmon and cavity photons is already well-studied. Future research directions lean toward the active and dynamic control of the polariton modes, e.g. by employing active MM or optically controlled MM. Following this line, an interesting direction is the study of non-Hermitian optics. By manipulating the losses and coupling strength of the coupled modes, one could reveal new features of such non-Hermitian optics. In the following, we discuss those fields of research on strongly interacting light–matter systems and provide perspectives of interesting new features.

**Figure 2:**
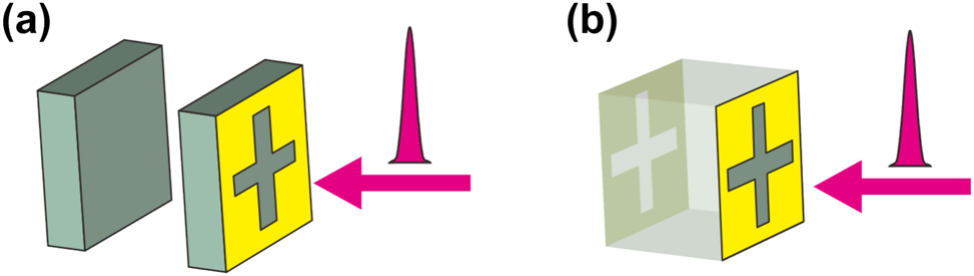
Schematics of the strongly coupled systems. (a) Schematic of a Tamm cavity loaded with a two-dimensional Swiss-cross CMM on the outer surface of a Bragg reflector (only one unit cell of MM is shown). (b) A Fabry–Perot cavity loaded with CMMs on both sides. In both (a) and (b), an ultrashort laser pulse selectively excites one CMM and switches off its plasmonic resonance.

A first such field of research is that of time-resolved spectroscopy, especially the investigation of sub-cycle dynamics of impulsively disturbed oscillating systems. Controlling the dynamics of the strongly coupled system has attracted significant interest recently [49]. Being able to switch on and off the polariton modes provides an important tool for the investigation of the interaction dynamics within the cavity, and knowledge of these processes is pivotal for the design of ultrafast optoelectronic devices. Two exemplary arrangements for such studies of polariton dynamics are shown in Figure 2. The version of Figure 2(a) is a so-called Tamm cavity consisting of a distributed Bragg reflector (in this case consisting of two dielectric slabs separated by an air gap) on top of a patterned or unpatterned metal layer [40]. Such cavities can support so-called Tamm states, electromagnetic modes which are localized at the interface between the Bragg reflector and the metal [50], [51]. In Figure 2(a), the metal structure is a terahertz Swiss-cross CMM. Being located on the outer surface of the Bragg reflector, it is accessible for external optical excitation. Upon irradiation with an intense (amplified) ultrashort optical pulse with a photon energy sufficient for interband excitation of the dielectric, a dense electron-hole plasma is created in the open areas of the CMM, electrically shorting the CMM and thereby efficiently switching off the CMM resonance within a time span of the pulse duration (typically on the 100-fs time scale) and much faster than the period of the cavity mode (which has a picosecond-scale duration). The rapid switching destroys the polariton modes [52]. How rapidly this occurs depends on the relative phase of the polariton oscillation at the moment of excitation, and is slower if the energy of the mode is in the radiation field at that moment rather than in the plasma oscillation. The detailed sub-cycle response shows rich interference dynamics strongly influenced by the initial strength of the mode coupling [49], [52], [53].

The study of sub-cycle polariton dynamics has only begun fairly recently [52]. There are many types of coupled systems waiting to be explored. An example of a hitherto unstudied arrangement is shown in Figure 2(b). It consists of a Fabry–Perot cavity with two CMMs replacing the conventional mirrors. Such a system allows for strong coupling among three quasi-particles: two plasmon modes and a cavity mode. Switching off of one CMM transforms the strong coupling of three quasi-particles into the strong coupling of two, converting the three-polariton system into one with two new polariton modes. The rich sub-cycle switching dynamics of such a system remains to be explored.

A second field of research where one can expect much future activity is that of active or reconfigurable MMs to be incorporated into cavities. Such MMs, which are highly interesting for applications such as light modulation or frequency-tunable optical filtering have undergone rapid development in recent years [54], [55], which led to the creation of versatile functional MM-based devices such as polarization manipulators and spatial light modulators [56], [57]. For the active reconfiguration of the MMs, different materials can be employed such as 2D materials [58] and semiconductors [54], [59], whose conductivity can be modified electrically or optically. In addition, reshaping of the geometrical structure of the MMs by micro-electromechanical-system (MEMS) components has also been adopted to actively control the resonance frequency of MMs [60]. It is promising to exploit these advancements by the integration of active MMs into cavities, thus enabling the active control of the coupling strength between MMs and cavity photons through external parameters.

Unconventional ways of external control are opened by the use of superconductors for the fabrication of the MM and/or the cavity. High-temperature superconductors were employed to develop MMs with resonant frequencies tunable over a wide THz frequency range. Superconducting elements can provide tunable electromagnetic properties, as they are sensitive to a variety of external disturbances such as temperature, magnetic field, electric current, and optical radiation. For example, high-Tc superconducting YBa_2_Cu_3_O_7_ split-ring resonators have recently been reported with tunability of the resonance frequency (up to 4 THz) by temperature [61]. Moreover, ohmic losses of superconductors are low up to a certain cutoff frequency and independent of the dimension of the component, which is highly advantageous for increasing the Q-factor of polariton modes [62].

A third development to be discussed is the transition from classical physics (coupling of various electromagnetic modes) to the quantum regime (coupling of electromagnetic modes to fermions) in combination with an increase of the coupling strength (from strong to ultrastrong and even deep-strong coupling). In the framework of solid-state cavity QED, polariton modes based on quantum-matter-loaded MMs ultra-strongly coupled to a cavity are very promising for the creation of novel types of hybrid states. The quantum matter can be a fairly simple fermionic system such as doped semiconductors at low-temperature where electronic transitions of the impurities are coupled to electromagnetic modes [63]. However, more complex arrangements are also of interest. A specific challenge is the coupling to spatially extended quantum matter such as an electron gas, e.g. in a single quantum well, exhibiting cyclotron oscillations and Landau level transitions in a magnetic field [7]. These are bosonic excitations and involve a large number of coupled electrons which are shared among many identical unit cells. The coupled light–matter system will be described by the bosonic Hopfield model where both material excitation and the electromagnetic field are boson fields [64]. There is currently a growing interest in studying ultra-strong light–matter coupled systems toward the limit of a few electrons coupled to a single cavity to study the fermionic Rabi model for the coupled systems [65]. To this aim, a challenge is to limit the in-plane spatial extension of the polariton modes so that the quantum system interacts with only a single MM unit cell coupled to the Fabry–Perot cavity. Limiting the in-plane spatial extension of the hybrid mode is also required for studying light–matter interaction with subwavelength-localized quantum systems, such as a quantum dot or a molecule. The hybrid modes confined in all directions of space will be made possible by reducing the transverse size of the Fabry–Perot cavity. One approach is to reduce the top metal layer of the Tamm cavity to a finite disk, as has already been achieved in the [66].

The fourth and final research direction which we want to address is that of non-Hermitian photonics. In quantum mechanics, the assumption of Hermiticity (operators are equal to their complex conjugate transpose) has traditionally been considered a fundamental requirement for Hamiltonians to exhibit real-valued eigenvalues. Lossless quantum systems, isolated from the environment, usually exhibit Hermiticity, open systems not. In that sense, all polaritonic systems, as considered in this publication, are strictly speaking open and non-Hermitian. However, recent developments in the context of open quantum systems have revealed that Hermiticity is not an absolute necessity for real eigenvalues. Certain non-Hermitian Hamiltonians, particularly those that respect parity-time (PT) symmetry, can also yield real eigenvalues [67]. The exploration of non-Hermitian Hamiltonians has experienced a substantial surge over the past decade, and novel concepts exploiting non-Hermiticity have been introduced to a plethora of quantum and classic systems, such as atoms, mechanical systems as well as optical ones [68], [69], [70], [71], [72].

With regard to non-Hermitian optics, intriguing and counterintuitive results have been reported. One of the most captivating features of non-Hermitian Hamiltonians is the presence of spectral branch-point singularities, known as exceptional points (EPs), where the real parts and imaginary parts of the eigenvalues coalesce when varying parameters such as coupling strength as well as loss and gain. These EPs mark the transition from the PT-symmetric to the PT-symmetry-broken phase. At the same time, they also mark the boundary between weak and strong coupling [73]. The simplest example of non-Hermitian optics is two coupled entities, such as two coupled resonators or two coupled (quasi-)particles. In this regard, the coupling of MMs and photonic cavities provides an appropriate platform for the investigation of non-Hermitian optics. Indeed, the strong coupling of MMs with photonic cavities can be well described by coupled-modes theory [30], [42]. The coupled-mode equations [42], [74] can be written as

(1) $$-j\frac{d}{dt}\left[\begin{array}{c} \Psi_{c}\\ \Psi_{m} \end{array}\right]=\left[\begin{array}{cc} \omega_{c}+j\gamma_{c} & V\\ V & \omega_{m}+j\gamma_{m} \end{array}\right]\left[\begin{array}{c} \Psi_{c}\\ \Psi_{m} \end{array}\right],\;\;$$

where Ψ_*c*_ and Ψ_*m*_ represent the respective field amplitudes of the cavity mode and the MM mode, *ω*_*c*_ is the angular eigenfrequency of the cavity mode, *ω*_*m*_ the angular resonance frequency of the MM, *γ*_*c*_ the photon decay rate (loss/gain) in the cavity, *γ*_*m*_ the damping rate (loss/gain) of the MM plasmons, and *V* the coupling parameter. The coupling matrix is non-Hermitian, and hence one expects to observe unique features of non-Hermitian optics in such a coupled system. By solving Eq. (1), one derives the eigenvalues

(2) $$\begin{array}{ll} \omega_{\pm } & =\frac{\omega_{c}+\omega_{m}}{2}+j\frac{\gamma_{c}+\gamma_{m}}{2}\pm \frac{1}{2}\cdot \\ & \;\sqrt{{\left(\omega_{c}-\omega_{m}\right)}^{2}-{\left(\gamma_{c}-\gamma_{m}\right)}^{2}+j2\left(\omega_{c}-\omega_{m}\right)\left(\gamma_{c}-\gamma_{m}\right)+4V^{2}}. \end{array}$$

These eigenvalues reflect the features of non-Hermitian optics. This is most easily seen, if we consider the special case that the resonance frequencies of the MM and the cavity coincide (*ω*_*c*_ = *ω*_*m*_), and that gain compensates the loss (*γ*_*c*_ = −*γ*_*m*_). In such a scenario, Eq. (2) reads

(3) $$\omega_{\pm }=\omega_{c}\pm \sqrt{V^{2}-\gamma_{c}^{2}}.$$

If $V^{2}>\gamma_{c}^{2}$ , one obtains two different real eigenvalues *ω*_±_. For this situation, it can be shown that PT symmetry is satisfied. In contrast, if $V^{2}<\gamma_{c}^{2}$ , the eigenvalues have the same real part, but different imaginary parts, leading the system into a PT-symmetry-broken phase. For $V^{2}=\gamma_{c}^{2}$ , only a single real eigenvalue exists, and one has a spectral singularity. This is what is called an EP.

While loss compensation (*γ*_*c*_ = −*γ*_*m*_) is imperative to establish PT symmetry [68], certain features of PT symmetry can be maintained with passive systems without active gain (quasi-PT symmetry). In this case without loss compensation, the eigenvalues of Eq. (2) are always complex-valued. If a global loss background is subtracted, one can demonstrate an effective gain that compensates for the residual loss [75].

**Figure 3:**
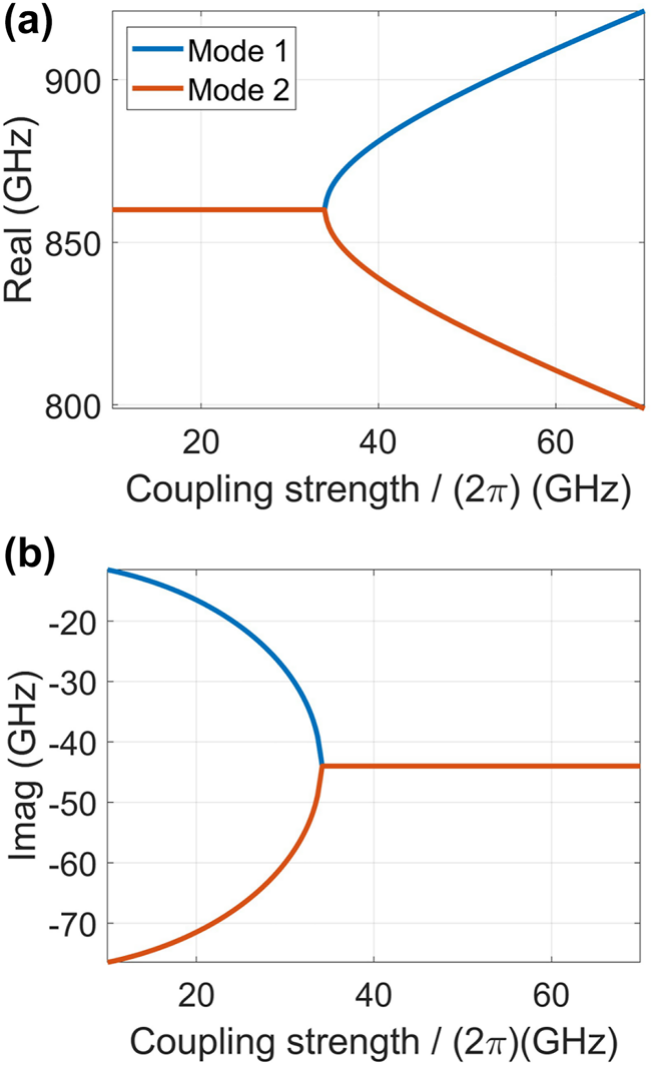
The real (a) and imaginary (b) parts of the eigenvalues of a two-coupled-mode system as a function of the coupling strength. Evaluation of Eq. (2) for the parameters of Ref. [30].

In an alternative scenario of passive systems, where the losses of the two coupled entities differ, an *effective* EP can be realized. We demonstrate this by evaluating Eq. (2) for the strongly coupled MM-cavity system of Ref. [30]. The relevant fixed parameters are *ω*_*c*_/(2*π*) = *ω*_*m*_/(2*π*) = 860 GHz, *γ*_*c*_/(2*π*) = 10 GHz, *γ*_*m*_/(2*π*) = 78 GHz, while the coupling parameter *V* is treated as a variable. Figure 3 displays the real and imaginary parts of the eigenvalues as a function of *V*. When $4V^{2}={\left(\gamma_{c}-\gamma_{m}\right)}^{2}$ , one obtains an EP. For weaker coupling, the eigenvalues have the same real part and differing imaginary parts, while for stronger coupling, the situation is reversed.

An EP has not been observed yet for MMs in a cavity. In systems such as those of Ref. [30], the losses are determined by the material and cavity properties, and one finds that the loss in the cavity is much smaller than that in the MM (*γ*_*c*_ ≪ *γ*_*m*_). The coupling strength is determined by the position of the MM in the cavity (mode overlap), as well as the net dipole moment of the MM which depends among other factors on the density of the unit cells of the MM. The design flexibility of both the MM and the cavity makes it principally convenient to tune the coupling strength to achieve the conditions for the observation of an EP. In addition, various other methods can be employed to continuously tune the coupling strength, e.g. by the use of reconfigurable MMs of which the effective dipole moment can be changed, or by employing MMs or CMMs on a semiconducting substrate which can be rendered electrically conducting by external optical excitation (cp. Figure 2(b)), thus tuning predominantly the loss of the MM.

The existence of EPs introduces exotic features to coupled systems, such as unidirectional reflection [75] and ultrasensitive sensing at the exceptional point [76]. The latter feature promises future sensors which allow tracing of minute amounts of analytes.

## 4 Conclusions

In this perspective, we have given a survey of the advances in the field of terahertz metamaterials strongly coupled to electromagnetic modes of photonic cavities. We have outlined potential future research directions. The coupling to photonic cavities provides not only new ways to manipulate and expand the properties of the metamaterials, but also brings forth novel features non-existing without the coupling, examples being the appearance of non-Hermitian optical properties, of novel features of many-particle interactions (bosonic and fermionic), and of complex sub-cycle dynamics upon ultrafast disturbance of the coupled system.
